# The accuracy of dental implant placement with different computer-assisted implant surgery techniques

**DOI:** 10.6026/973206300221409

**Published:** 2026-03-31

**Authors:** Sindhu Sudhakar Kumararama, AB Nischitha, H Sneha, Bhavna Jha Kukreja, Md Jalaluddin, Ripal Parikh, Miral Mehta

**Affiliations:** 1 Department of Prosthodontics, Crown & Bridge, Rajarajeswari Dental College and Hospital, Bengaluru, Karnataka, India; 2 Department Preventive Dental Sciences, Gulf Medical University, Ajman, UAE; 3 Department of Periodontics and Implantology, Kalinga Institute of Dental Sciences (KIDS), Campus-5, KIMS, KIIT Deemed University, Bhubaneswar-751024, Odisha, India; 4 Department of Periodontics, Dharmsinh Desai University, Gujarat, India; 5 Department of Pediatric and Preventive Dentistry, Karnavati School of Dentistry, Karnavati University, Gandhinagar, Gujarat, India

**Keywords:** Dental implants, computer-assisted surgery, static surgical guide, dynamic navigation, implant accuracy

## Abstract

Despite technological advances, achieving ideal three-dimensional precision in dental implant placement remains challenging, with deviations potentially affecting prosthetic outcomes and peri-implant health. Therefore, it is of interest to evaluate the accuracy of static computer-guided, dynamic computer-guided and conventional freehand implant placement techniques. Hence, a total of 120 implants were placed in 96 patients, with at least 40 implants per group and postoperative CBCT images were compared with preoperative plans to measure positional deviations. Both computer-guided approaches demonstrated significantly higher placement accuracy than the freehand method (p < 0.05). Dynamic navigation achieved the best accuracy with minimal coronal (0.71 ± 0.34 mm) and angular (2.18 ± 1.12°C) deviations, indicating its slight advantage in clinical predictability over static guidance.

## Background:

The use of dental implant therapy has become the standard of care when utilising the procedure as a replacement for missing teeth, providing predictable functional and aesthetic outcomes with high long-term survival rates [1]. Implant treatment is not only with regard to the success in the process of osseointegration, but also with regard to the perfect positioning in three-dimensionality, which guarantees the best prosthetic restoration, distribution of loads and maintenance of neighbouring anatomical structures [2]. Inappropriate placement of the implant may result in biomechanical difficulties, aesthetic issues, bone loss around the implant and, in the worst scenario, injury to the vital structures like the inferior alveolar nerves or the maxillary sinus [3]. Conventional methods of implant placement are based on the clinical experience of the surgeon, his/her anatomical understanding and the evaluation of radiographs (2D). Although skilled clinicians may produce satisfactory results using such a method, it has been shown that there is a high degree of variability in the placement of implants with angular deviations of more than 10 degrees and linear deviations of over 2mm recorded in significant percentages of cases [4]. These errors prove especially troublesome in situations where the bone volume is limited; there are several neighbours of implants or immediate loading programs that need an exact linkage between the prosthetics [5]. Computer-assisted implant surgery (CAIS) has become a technology that enhances the accuracy of placement and predictability of the treatment. They are systems based on cone-beam computed tomography (CBCT) data, which are combined with dedicated software to allow performing the virtual planning of the implants based on anatomical and prosthetic demands [6]. The virtual plan may then be transferred to the surgical field by use of either the static or dynamic guidance system, with each having different pros and cons [7]. Computer-guided surgery Static Computer-guided surgery uses stereolithographic surgical templates produced by computer-aided design and manufacturing (CAD/CAM) technology. These guides include metal sleeves that literally guide the drilling process and the insertion of the implants along the pre-established path [8]. Mean deviation of the entry point and apex has been reported to be about 1.0-1.2 mm and 1.2-1.5 mm, respectively and the angular deviation is 3-4 degrees [9]. Although these accuracy rates are a substantial advance over freehand surgery, there are fundamental limitations of template-based guidance, such as limited visualisation, contraction to open the mouth that can be solved and inability to alter the plan during surgery [10]. The other method is dynamic navigation that would give real-time feedback of the position of the surgical hand piece in view of both the anatomy of the patient and the virtual plan. The surgeon is able to see the drill path on a screen and make corrections during the procedure using optical or electromagnetic tracking systems [11]. This technology does not require physical surgical guides and has the flexibility to allow intraoperative changes. Recent studies have shown precision equivalent to or even greater than that of a static direction and some studies have shown that the deviations of the mean are less than 1 mm at both coronal and apical levels [12]. A consistent body of comparative research on the effectiveness of static versus dynamic guidance in relation to specific systems being tested, the operating room and the experience level of the operating room has produced inconsistent results. The meta-analyses have indicated that the two methods have adequate accuracy to be used in clinical practice, but direct comparisons in the same study population are still lacking [13]. In addition, the majority of current studies have been done on fully edentulous or cadaver models and there have been limited studies on the accuracy of partially edentulous patients when anatomy-related limitations and prosthetic requirements may be different [14]. The learning curve of the various CAIS technologies is a significant practical aspect. The implementation of guided surgery in the static mode requires the mastery of digital planning and knowledge of the principles of template design and in the dynamic mode; it needs the coordination between the visual feedback and motor skills in real-time [15]. Comparison of the accuracy development as experience is gained could be of great importance to clinicians who embrace such technologies [16]. In spite of the growing body of research on computer-assisted implantology, there are still doubts about the relative efficiency of the existing technologies when used in similar clinical settings. Comparisons between fixed instructions, dynamic navigations and freehand placements in one group of patients on a standardised measurement protocol would be required to present evidence-based suggestions concerning the choice of technique. Therefore, it is of interest to measure and compare the accuracy of dental implant placement between using the stagnant computer-guided surgery, dynamic navigator surgery and the traditional freehand technique on partially edentulous patients.

## Materials and Methods:

## Design of the study and ethical concerns:

The study was a prospective comparative clinical trial, which was carried out at the Department of Oral and Maxillofacial Surgery of one of the university dental hospitals between March 2022 and August 2024. The Institutional Review Board gave its consent to the study protocol and all the procedures were conducted in line with the Declaration of Helsinki. All the participants signed the informed consent written before enrollment.

## Sample size calculation:

The sample size was calculated depending on earlier research that indicated the differences in linear deviation of the guided and freehand placement of implants. The sample size was determined by assuming a difference in mean of 0.5 mm between the groups, a standard deviation of 0.6 mm, AE of 0.05 and a power of 85, which indicated that the required sample size was 35 implants in each group. Since there was a possibility of loss of data or missing measurements, 40 implants were to be used in each group, which included 120 implants altogether.

## Patient selection:

Eligibility screening of patients was done who had to have single or multiple implant placements in partially edentulous arches. The assignment of treatment groups was executed in such a manner that a computer-generated randomisation sequence with an allocation concealed.

## Inclusion criteria:

[1] Age 18 years or older

[2] Sufficient bone volume to place the implants without grafting at the same time.

[3] Single or more confined edentulous spaces.

[4] Adequate mouth opening (≥35 mm)

[5] Good general health status

[6] Ready to abide by research procedures.

## Exclusion criteria:

[1] Uncontrolled systemic illnesses (diabetes HbA1c > 8%, immunocompromising problems)

[2] Active periodontal disease

[3] History of bisphosphonate treatment or radiation of the head and neck area.

[4] Smokes more than 10 cigarettes per day.

[5] Pregnancy or lactation

[6] Lack of bone that necessitates bone augmentation operations.

[7] Fully edentulous arches

[8] Patients who have movement disorders related to the precision of surgery.

## Treatment groups:

Samples were randomly chosen to belong to one out of three treatment groups:

[1] Group A (Static Guided Surgery, n=40 implants): Implants set in place with the help of the stereolithographic surgical templates created in accordance with the virtual planning, which was built on CBCT.

[2] Group B (Dynamic Navigation Surgery, n=40 implants): Implants with an optical navigation system that tracked intraoperatively and used a real-time optical navigation system.

[3] Group C (Freehand Surgery, n=40 implants): Implants were positioned with the help of the traditional freehand method under the 2D radiographic control.

## Preoperative planning:

Each patient was imaged with a preoperative CBCT imaging (Planmeca ProMax 3D Mid, Planmeca Oy, Helsinki, Finland) using standardised acquisition settings (90 kV, 10 mA, 0.2 mm voxel size). CBCT data of Groups A and B were then imported into implant planning software (coDiagnostiX, Dental Wings, Montreal, Canada). An intraoral scanner (TRIOS 3, 3Shape, Copenhagen, Denmark) was used to obtain the digital impressions, which were then combined with the CBCT data to create integrated anatomical-prosthetic models. The planning of virtual implants was done by one experienced operator based on the availability of bones, the need for the prosthetic and the proximity to the vital structures. The dimensions of the implants (diameter 3.5-4.5 mm, length 8-13 mm) were determined by the available bone requirements and the prosthetic requirements.

[1] In Group A, surgical guides were made in the form of tooth-supported models having fully guided guidelines. Manufacture of guides was done through stereolithography (Form 3B, Formlabs, Somerville, MA) with biocompatible surgical guide resin (Dental SG Resin, Formlabs). Metal sleeves (2.0 mm inner diameter) were added in accordance with the intended positions of the implants.

[2] In the case of Group B, the virtual plan was transferred to a dynamic navigation system (Navident, ClaroNav, Toronto, Canada) to be used as intraoperative guidance.

[3] In the case of Group C, conventional two-dimensional treatment planning was done using panoramic radiographs and periapical radiographs.

## Surgical procedure:

All operations were conducted by two senior oral surgeons (more than 5 years of experience with implants, more than 50 guided surgeries each), using local anaesthesia. The same implant system was used (Straumann BLT, Straumann AG, Basel, Switzerland).

[1] Group A- Static Guided Surgery: Template verification and seating were done, followed by sequential drilling through the guide sleeves as per the instructions of the manufacturer. The insertion of the implants was done via the guide in a torque-controlled manner.

[2] Dynamic Navigation (Group B): The navigation system was calibrated after registering the position of the patient with the help of fiducial markers. The presence of real-time tracking made it possible to visualise drill position and drill trajectory on the display screen. The surgeon carried out osteotomy and placement of implants as he was observing the deviation of the intended position.

[3] Freehand Surgery (Group C): Mucoperiosteal flap rising was done conventionally and implant placement was done based on clinical judgment, anatomic landmarks, as well as reference to two-dimensional radiographs.

They all had primary stability (insertion torque 25 Ncm and above). The resorbable sutures were used in wound closure.

## Postoperative assessment:

CBCT images were also acquired within 1 week of surgery under the same acquisition conditions as those of preoperative imaging. The DICOM data were imported and superimposed on the preoperative planning datasets on the analysis software (coDiagnostiX) after they were imported as postoperative data.

The prior blinded investigator measured the following parameters of deviation:

[1] Coronal (Platform) Deviation: 3-dimensional linear distance between planned and actual implant platform centres (mm)

[2] Apical Deviation: Linear displacement of the planned and actual apex of implants (3D, mm) between planned and actual positions.

[3] Angular Deviation: Angle of planned to actual implant axes (degrees)

[4] Depth Deviation: Vertical disparity of planned and actual positions of implants in the implant axis (mm)

Two measurements were conducted after 2 weeks and the analysis was done using mean values. To determine intra-observer reliability, the intra-class correlation coefficient (ICC) was used.

## Statistical analysis:

The SPSS version 27.0 (IBM Corporation, Armonk, NY) was used in the analysis of data. Continuous variables were reported as mean, SD, median and range. The Shapiro-Wilk test was used to test the normality. The accuracy parameters of three groups were compared through a one-way analysis of variance (ANOVA) on the normally distributed data, which was then followed by the Tukey post hoc test to make pairwise comparisons. Non-normally distributed variables were analysed by using the Kruskal-Wallis test. The effects of the location of implants (anterior versus posterior, maxilla versus mandible) and surgical experience on the results of the accuracy were tested by subgroup analysis. Several linear regressions were conducted to establish the factors that deviate. The cut-off point of statistical significance was p < 0.05.

## Results:

A total of 120 dental implants were placed in 96 patients (58 males, 38 females) with a mean age of 52.4 ± 12.8 years. The distribution of implants across groups and their characteristics are presented in Table 1 (see PDF). No significant differences were observed among groups regarding patient age, gender distribution, implant location, or implant dimensions (p > 0.05). Intra-observer reliability for deviation measurements demonstrated excellent agreement (ICC = 0.94-0.97 for all parameters). All implants achieved primary stability and no intraoperative complications were recorded. The accuracy parameters for each treatment group are summarised in Table 2 (see PDF). Significant differences were observed among groups for all deviation measurements (p < 0.001).Mean coronal deviation was lowest in the dynamic navigation group (0.71 ± 0.34 mm), followed by static guided surgery (0.89 ± 0.41 mm) and highest in the freehand group (1.54 ± 0.62 mm). Post-hoc analysis revealed significant differences between computer-assisted techniques and freehand placement (p < 0.001), while the difference between static and dynamic navigation approached but did not reach statistical significance (p = 0.067).Similar patterns were observed for apical deviation, with dynamic navigation achieving the lowest mean deviation (0.94 ± 0.42 mm), compared to static guided (1.18 ± 0.53 mm) and freehand (1.89 ± 0.71 mm) techniques. All pairwise comparisons were statistically significant (p < 0.05). Angular accuracy was significantly superior with dynamic navigation (2.18 ± 1.12°C) compared to both static guided (2.87 ± 1.45°C) and freehand (4.92 ± 2.34°C) approaches. The difference between dynamic navigation and static guided surgery was statistically significant (p = 0.024).Depth control was most precise with dynamic navigation (0.42 ± 0.28 mm), followed by static guided (0.58 ± 0.34 mm) and freehand (0.89 ± 0.51 mm) techniques. Subgroup analyses examining the influence of anatomical location and jaw on accuracy outcomes are presented in Table 3 (see PDF). Within each treatment group, no significant differences were observed between maxillary and mandibular implants for any deviation parameter (p > 0.05). However, a trend toward higher angular deviation was observed for posterior implants compared to anterior implants across all groups, though this difference reached statistical significance only in the freehand group (5.34 ± 2.48°C vs. 4.22 ± 1.98°C, p = 0.042). Multiple linear regression analysis identified treatment group (β = -0.412, p < 0.001) and implant length (β = 0.187, p = 0.018) as significant predictors of apical deviation. Longer implants were associated with greater apical deviation across all groups. Patient age, gender and implant diameter were not significantly associated with deviation parameters.

## Discussion:

The given study is a full-scale comparative analysis of the accuracy of implant placement with the use of the following methods: static computer-guided surgery, dynamic navigation and the traditional freehand approach in the partially edentulous group of patients. The two computer-aided techniques indicated much better precision than the free-hand positioning in all of the parameters measured, with the dynamic navigation approaches having marginally better angular precision. The average coronal deviation of 0.89 mm with the use of stationary guided surgery in this paper is in accordance with the published systematic reviews, with a range of 0.9 to 1.2 mm [17]. Equally, the angular deviation of 2.87 degrees is within the range anticipated in template-guided placement of implants. These results validate the accuracy and repeatability of static guidance, on the condition of being well planned and with sufficient protocols for template fabrication. Dynamic navigation showed a slight improvement in accuracy over static guidance and statistically significant variations were found in angular deviation. Its angular deviation of 2.18 degrees and a coronal deviation of 0.71 mm with dynamic navigation are consistent with other recent clinical studies, which have shown high accuracy of real-time tracking systems [18]. The enhanced angular control can be explained by the constant display of the angular feedback during the drilling process, which enables the real-time correctional changes that could not be applied in the case of template guidance, which is rigid [19]. The benefit of dynamic navigation in accuracy might be especially useful in anatomically challenging positions in which accurate angulation is essential to avoid important structures or maximise the emergence of the prosthetic. Past studies have shown that dynamic systems can be used to reliably place even in situations with a small interocclusal space or limited access, allowing the use of standard surgical templates to be avoided [20]. The freehand technique, although showing satisfactory results in a skilled hand, showed much more deviation values in all parameters. The average angular error of 4.92 degrees and coronal error of 1.54 mm highlight the shortcomings of clinical judgment decisions and two-dimensional imaging in determining the implant position. These results can be compared with the existing literature that reports the imprecision of the freehand placement, especially in less experienced operators [21]. The clinical value of the observed deviations should be considered. Although statistical differences were actually observed between groups, it is a question of whether such differences result in any clinically relevant information or a clinical outcome that relies on the specific case requirements. The range of 1-2 mm can be acceptable in situations where there is plenty of bone and other factors are favourable to prosthetic placement, but problematic in situations where immediate loading is needed, or the implant placement is adjacent to other structures [22]. The fact that there is no statistically significant difference in accuracy between maxillary and mandibular implants implies that differences in bone density do not have a significant effect on the accuracy that can be attained when using computer-assisted techniques. Nevertheless, the movement towards the escalating angular deviation in the posterior parts might be connected with the higher complexity of the technique to get the optimal access and visibility in those parts [23].

The impact of the implant length on apical deviation, which was found during the regression analysis, has a practical implication on treatment planning. The longer the implants, the more apical they tend to deviate upon any given angular error and therefore it is important that the angular control must be accurate, once long implant lengths are used. This mathematical connection is to be taken into consideration during the placement of implants near the inferior alveolar canal or maxillary sinus floor [24]. Practically, either of the two computer-aided methods entails extra expenses and workflow implications for the freehand placement. Guided surgery involves the use of templates (static guided surgery), which typically increases the cost of treatments by 1-2 weeks and necessitates a laboratory cost. Dynamic navigation does not require templates but requires the purchase of navigation equipment and calibration during surgery [25]. Another significant factor to keep in mind when accept these technologies is the learning curve of each technique for clinicians. Research has proven that dynamic navigation has a learning curve of between 10 and 20 cases before maximum accuracy is reached and static guided surgery can have a slightly shorter learning curve since it has physical limitations in the form of the template [26]. In this study, procedures have been done by trained operators and this might not be generalizable to new users. More recent studies have addressed the precision of fully guided implant placement as compared to partially guided protocols, whereby only the osteotomy is template-directed with implant insertion at freeland. There is some evidence that full protocol guidance is more accurate, but possibly at the expense of less tactile information during insertion [27]. Integration of intraoral scanning and CBCT data to perform virtual planning has simplified the digital workflow and could lead to increased accuracy, which may be due to the fact that there may be no error that could be created by the traditional methods of impression. The visualisation of the hard and soft tissue relationship in the planning software allows a more accurate positioning as compared to the planned outcome of the prosthetic [28]. There are various limitations that should be acknowledged in this study. The small sample size in each subgroup restricts the statistical power in the detection of differences in the anatomical location analyses. External validity may be limited because of the single-centre design and participation of experienced operators. Also, the clinical outcomes, like the fit of the prosthetics, aesthetic outcomes and survival of the implants, were not analysed in this study. Long-term comparative outcomes, such as the implant survival rates, marginal bone alterations and complications of the prosthetics, should be studied in the future to identify whether the accuracy benefits of computer- assisted methods can be translated into better clinical performance. Also, cost-effectiveness studies that would include equipment prices, workflow productivity and complication rates would be helpful in clinical decision-making.

## Conclusion:

Both static and dynamic computer-guided implant surgeries demonstrated markedly superior accuracy compared to the conventional freehand approach, with dynamic navigation showing statistically significant advantages in angular precision. The mean deviations observed in both guided techniques remained within clinically acceptable limits, supporting their routine clinical use for predictable implant placement. Computer-assisted implant surgery can thus be considered the emerging standard of care, though further long-term studies are needed to assess its impact on clinical and prosthetic outcomes.
